# Mortality in homeless people enrolled in the French housing first randomized controlled trial: a secondary outcome analysis of predictors and causes of death

**DOI:** 10.1186/s12889-021-11310-w

**Published:** 2021-07-02

**Authors:** Aurélie Tinland, Sandrine Loubiere, Matthieu Cantiello, Mohamed Boucekine, Vincent Girard, Owen Taylor, Pascal Auquier

**Affiliations:** 1 Department of Psychiatry, Marseille Public Hospital, 147 boulevard Baille, F-13005 Marseille, France; 2 Aix-Marseille University, School of medicine – La Timone Medical Campus, EA 3279: CEReSS – Health Service Research and Quality of Life Center, F-13005 Marseille, France; 3 Department of Clinical Research and Innovation, Support Unit for clinical research and economic evaluation, Marseille Public Hospital (APHM), F-13385 Marseille, France

**Keywords:** Mortality, Homelessness, Housing first, Schizophrenia, Bipolar disorders, Health inequalities

## Abstract

**Background:**

Homeless people face large excess mortality in comparison with the general population, but little is known about the effect of housing interventions like Housing First (HF) on their mortality.

**Aims of the study:**

1) to explore 2-years mortality among homeless people with severe mental illness (SMI) included in French HF randomized controlled trial (RCT). 2) To examine causes of death among homeless participants.

**Methods:**

For 703 participants of HF RCT: 353 in experimental group (HF) and 350 in control group (Treatment As Usual - TAU), any proof of life or death and causes of death were collected with a thorough retrospective investigation among relatives, institutions and administrative databases. Data collection took place from March to June 2017.

**Results:**

4.8% (*n* = 34) of the study participants died over the study period. Mean age of death was 40.9 (+/− 11.4) years. The overall 2-years mortality rate was 0.065 in the HF group (*n* = 23) versus 0.034 in the TAU group (*n* = 11). Mortality was associated with medications for opioid use disorder in multivariate Cox analysis (HR: 2.37, 95%CI 1.15–5.04, *p* = 0.025). Those in HF group seem to be more at risk of death compared to TAU group, mainly during the first 6 months of being housed, although the difference did not reach significance (HR: 0.49, 95%CI 0.24–1.01, *p* = 0.054). Violent deaths occurred in 52.2% of HF group’s deaths versus 18.2% of TAU group’s deaths, this excess being explained by 34.8% (*n* = 8) deaths by overdoses in HF group versus none in TAU group.

**Limitations:**

1) 8.7% (*n* = 2) people in HF group died before HF intervention but were analyzed in intention-to-treat. 2) No proof of life or death has been found for only 0.6% in HF group (*n* = 2) but for 9.5% people in TAU group (*n* = 33) that could be anonymous deaths. 3) Undetermined causes represented 8.7% of deaths in HF group versus 36.4% in TAU group. 4) The small number of events (deaths) in the study population is a limitation for statistical analysis.

**Conclusions:**

Due to important limitations, we cannot conclude on HF effect on mortality, but our results nevertheless confirm that the vulnerability of long-term homeless people with SMI persists after accessing independent housing. Earlier intervention in the pathways of homelessness should be considered, alongside active specific support for addictions.

**Trial registration:**

Ethics Committee Sud Mediterrannée V n° 11.050: trial number 2011-A00668–33: 28/07/2011. Clinicaltrials ID NCT01570712: 4/4/2012.

## Background

People who experience homelessness have an excess mortality rate three to ten times higher than that of the general population [1–3]. Both somatic and mental illnesses have been shown to be predictors of mortality among people experiencing homelessness, among other social indicators [2, 4–8]. Studies from Sweden and Denmark have shown a particular increase of mortality risk associated especially with substance use disorders and dual diagnosis with other mental illnesses [9–11]. These statistics are affected by the large amount of missing data in this area, and particularly a lack of routinely recorded data on homelessness in death records [4].

Moreover, information on causes of deaths in the homeless population is sparse, with a much higher rate of deaths due to unknown or unclear causes compared to the general population (28% compared to 9% after standardization on age and gender in France) [12]. This both limits adequate understanding of the leading causes of excess mortality among people experiencing homelessness, and at the same time indicates a disturbing level of social disconnection and indifference [13].

The *Un Chez Soi d’Abord* trial presented the opportunity to gather data on a number of people who had experienced of long periods of homelessness and to compare the effects of the Housing First (HF) intervention to a Treatment as Usual (TAU) group as part of a randomized controlled trial [14]. The results of the French HF study extended previous studies with respect to housing stability [15–20], demonstrating the HF effectiveness in helping individuals to escape homelessness by achieving housing stability while decreasing days spent in hospital [14]. By looking at mortality rates within these groups it is possible to make inferences about the impact of secure housing on mortality rates for people who were homeless and who suffered from severe mental illness and/or addiction problems. From these data we can begin to formulate a tentative analysis of the persistence of excess mortality in homeless population after access to housing.

The aim of the present study was then to explore a secondary outcome: mortality among homeless adults, and examine the role of the HF program on all-cause mortality, using data from “*Un Chez Soi d’Abord”* study [21]. Its secondary objectives were to examine causes of death among HF participants.

## Methods

### Trial design

*Un Chez Soi d’Abord* is a randomized controlled trial (RCT) enrolling homeless adults with severe mental illness in 4 large cities in France: Paris, Marseille, Toulouse, and Lille. Study participants were randomized 1:1 to the HF or TAU groups from August 2011 to April 2014 and followed over a 2-year period, with follow-up interviews conducted every 6 months. Details of the RCT protocol (including randomization and sample size calculation) have previously been described [21]. The relevant institutional review boards approved the trial on 28/07/2011 (Ethics Committee, trial number 11.050 and the French Drug and Device Regulation Agency) and the trial was registered in France under the number 2011-A00668–33. The registration on the site of Clinicaltrials was made retrospectively on 040/4/2012 with number NCT01570712.

### Participants

Participants were recruited from homelessness shelters, mobile outreach teams, community mental health teams, hospitals, and prisons. Eligible participants were adults over 18 years of age, with severe mental illness defined as schizophrenia (SCZ) or bipolar disorder (BD) diagnosis according to the Diagnostic and Statistical Manual of Mental Disorders, fourth edition (DSM-IV-TR) [22], being absolutely homeless (i.e. no fixed place to stay for at least the previous 7 nights with little likelihood of finding a place in the upcoming month) or precariously housed (i.e. housed in a single room occupancy, rooming house or hotel/motel as a primary residence AND with history of two or more episodes of being absolutely homeless in the past year OR one episode of being homeless for at least 4 weeks in the past year). Inclusion criteria also included moderate-to-severe disability according the Multnomah Community Ability Scale (MCAS) (score ≤ 62; range 17–85) [23], and at least one of the following criteria: (i) ≥2 hospitalizations for mental illness over the last 5 years; (ii) comorbid alcohol or substance use disorder; (iii) having been arrested or incarcerated over the previous 2 years. In addition, patients were required to be covered by French state health insurance, to have lived in the city concerned for over 6 months and intended to stay in that city for the next 2 years.

### Interventions

In the HF group, participants were offered scattered-site housing after their inclusion. They had some choice in the location and type of housing. They paid a maximum of 30% of their income as rent, depending on their resources, with the rest paid by the program (through the rent intermediation system). Individuals were firstly subtenants of their flat, becoming thereafter tenants through a lease transfer when they had sufficient resources. According to the HF model for a high level of needs, the multidisciplinary support teams (social worker, nurse, doctor, psychiatrist, and peer worker) followed an Assertive Community Treatment (ACT) model, with a recovery-oriented approach. It operated with a 10:1 client-staff ratio. Participants were provided with at least one weekly visit either at home or in the city at times convenient to them. Compliance with the recommendations for implementing the HF model of the U.S. authors was verified at each stage using the HF Model fidelity scale.

In the TAU group, homeless individuals received usual care, namely pre-existing dedicated homeless-targeted programs and services, including but not limited to outreach teams, shelters, and day-care facilities. Existing TAU services in France are numerous but heavily compartmentalized between housing and health services. In addition the French TAU’s system for social integration does not offer direct access to housing. These standard services mostly use a gradued approach where access to transitional housing is conditioned by abstinence and compliance with psychiatric treatment.

### Data collection

From March to June 2017 (i.e. at least 24 months for the last participant enrolled), we collected specific data to explore the issue of mortality in depth.

- Any proof of life or death and causes of death: a thorough retrospective investigation was carried out among family, friends, social and medical institutions, HF and outreach teams, and administrative databases. For the latter, several sources of information were investigated: the social emergency call center database (Samu social), hospital records, the French social security system database, non-governmental organizations databases (NGOs) like ‘Morts de la Rue’ (Dead on the Streets), and civil registries of the place of birth and known residence for those who were lost to follow-up. A resident doctor was in charge of collecting data and to assist the interviewers involved in the French randomized trial.

“Uncertain vital status” was defined by the absence of any evidence of life or death from all sources (families, friends, local institutions or administrative databases).

Death certificates were collected for all deceased people. These legal documents are written by medical practitioner, who fill in the date and cause(s) of death. They are linked to administrative databases.

The use of “internal” and “external” categories for causes of death refers to the *International Classification of Diseases, Tenth Revision*, where external causes include intentional and unintentional injury, poisoning including drug overdose (identified with codes S00 to T98, and V01 to Y98).

The following outcomes were assessed at different times between baseline and 24 months:

- Social functioning score at baseline assessed using the MCAS [23]. The MCAS is a 17-item instrument that measures the degree of functional ability of adults who have severe and persistent mental disorders and live in the community. Higher scores indicate more severe disability.
- Perceived physical and mental quality of life (QoL) assessed using the Medical Outcomes Study 36-item Short Form Health Survey (SF-36) [24, 25]. Eight dimensions are described: physical functioning, social functioning, role-physical problems, role-emotional problems, mental health, vitality, bodily pain, and general health. Two composite scores are calculated, the physical composite score (PCS) and the mental composite score (MCS), ranging from 0 (lowest QoL), to 100 (highest QoL). This outcome was assessed every 6 months between baseline and 24 months.
- Substance and alcohol dependences assessed using sections K and J of the Mini International Neuropsychiatric Interview (MINI) [26], an abbreviated, structured diagnostic interview that determines the presence or absence of diagnoses of dependence on and/or abuse of the more frequently used or more problematic drugs. Those outcomes were assessed at baseline, 12 and 24 months.
- Medicine information: use of medicines (name and dose of the medication actually taken) were assessed using self-reported data for the 6-month period preceding the evaluation. This outcome was assessed at baseline, 12 and 24 months.
- Utilization of health services, measured by the number of hospitalizations and length-of-stay for each hospitalization, was based on patients’ self-report data for the 6-month period preceding the evaluation. Those outcomes were assessed every 6 months between baseline and 24 months.
- Sociodemographic information at baseline: gender, age, education level, duration of lifetime homelessness, and number of nights spent homeless over the past 6 months using a retrospective calendar. The number of nights spent homeless were recoded into the categories of the European Typology of Homelessness and Housing Exclusion [27], which describe all living situations, from sleeping in public spaces to living in extremely over-crowded spaces.

### Statistical analysis

Data are presented as frequencies and percentages for categorical data and median ± standard deviation (SD) for continuous data. Significant differences between groups in demographic characteristics and secondary outcomes were examined using a chi-square test or Fisher’s exact test for categorical data and Student’s *t*-test or non-parametric test for continuous data, as appropriate. All *p* values were two-tailed and *p* values < 0.05 were considered as significant.

Kaplan-Meier methods along with the log rank and Wilcoxon tests were used to establish statistical differences in survival between the two groups and estimated mortality rates at 24 months. Survival was calculated as the number of days from the date of inclusion to participant’s death due to any given cause. Patients still alive were censored at the date of the last follow-up visit or the date of the last news for those who were initially lost to follow-up. The cut-off date was 24 months after enrolment for each participant.

Cox model with time-dependant covariates were used to assess the influence of covariates on the mortality rate, with “group” as the independent variable. Due to missing data in covariates, multiple imputation approach was performed (i.e. 100 imputed data sets) [28]. Imputation models were implemented using MICE by chained equations and mitools R packages. Potential factors affecting survival were investigated in univariate analyses. The continuous skewed covariates, namely hospitalisations and medicines, were transformed into categorical variables based on the median value. In addition, we investigated separately the impact of medications for opioid use disorder (MOUD), psychotropic treatment use and other treatments use as categorical variables (Yes/No). Variables meeting a threshold of *P* < 0.05 in univariate analysis were included in the multivariate analysis. All interactions between variable “group” and covariates were tested and only those that were significant were kept. Immortal time bias was not observed in our RCT design as no statistically significant differences were observed in lengths of time since homeless and mean age between the two groups [29].

Causes of death among participants were compared between the two groups of randomization: HF and TAU. Causes of death were classified as natural, violent (homicide, suicide, accidental), or undetermined. Among natural causes, the primary medical cause of death was noted when specified (e.g., infectious, cardiovascular or liver diseases, and cancers), and the term ‘other natural death’ was reserved for deaths following unspecified disease. In the category of violent deaths, overdoses of drugs were not included in accident.

SPSS version 20.0, and RStudio version 3.2.1 statistical software were used for statistical analyses.

## Results

### Characteristics of the study sample

A total of 703 homeless individuals were included in this study: 353 were assigned to the HF group and 350 to the TAU group. Among people allocated to HF, 16 did not receive allocated intervention. The mean age of the whole sample was 38.8 years (± 10.0), 82.6% were men and 68.9% were diagnosed with schizophrenia disorders (Table 1). The median duration of homelessness was 6 years (interquartile range: 2–12 years). During the 6 months preceding the enrolment in the study, the mean number of nights on the streets was 53 (± 68). Sociodemographic and clinical characteristics were compared between HF and TAU groups (Table 1). Briefly, when comparing gender, mean age, severe mental illness, education level and duration of homelessness, no differences were observed between the two groups. However, participants in the HF group were more absolutely homeless (*p* = 0.04), took more other treatment (*p* = 0.004) and had more alcohol dependence (*p* = 0.02) than those in the TAU group.

**Table 1:** Baseline Characteristics of Participants (*N* = 703)

Characteristics	Total (*n =* 703)	HF Group (*n* = 353)	TAU Group (*n* = 350)	*p*-value
Gender, No. (%)				0.10
Men	580 (82.5)	283 (80.2)	297 (84.9)	
Women	123 (17.5)	70 (19.8)	53 (15.1)	
Age mean (SD), y	38.7 (10.0)	38.1 (9.7)	39.4 (10.3)	0.09
Education, No. (%)				0.52
Less than high school (<bac)	490 (73.0)	249 (71.9)	241 (74.1)	
Completed or postsecondary school	181 (27)	97 (28.0)	84 (25.8)	
Housing status, No. (%)				**0.04**
Precariously housed	238 (34.0)	107 (30.3)	131 (37.6)	
Absolutely homeless	463 (66.0)	246 (69.7)	217 (62.3)	
Lifetime duration of homelessness, Median (IQR), months	72.0 (33–144)	72 (24–144)	72 (30–144)	0.99
Mental disorder, No. (%)				0.81
Schizophrenia	487 (69.3)	243 (68.8)	244 (69.7)	
Bipolar	216 (30.7)	110 (31.1)	106 (30.2)	
CGI Score mean (SD)	4.6 (1.3)	4.6 (1.3)	4.6 (1.2)	0.64
MCAS score mean (SD)	51.1 (7.2)	51.2 (7.5)	51 (7.0)	0.20
PCS SF-36 score mean (SD)	50.1 (11.6)	50.3 (10.7)	50.2 (12.0)	0.610
MCS SF-36 score mean (SD)	34.6 (10.0)	34.8 (9.8)	34.4 (10.2)	0.497
Violent victimization, No. (%)	225 (33.3)	116 (33.2)	109 (33.4)	0.957
Nonviolent victimization, No. (%)	356 (52.7)	190 (54.4)	166 (50.9)	0.360
Hospitalization, No. (%)	408 (60.8)	215 (61.8)	193 (59.8)	0.591
Taking medicines, No. (%)	513 (73.0)	263 (74.5)	250 (71.4)	0.359
MOUD	136 (19.3)	64 (18.1)	72 (20.6)	0.413
Psychotropic drugs	473 (64.3)	241 (68.3)	232 (66.3)	0.575
Other treatments	55 (7.8)	38 (10.8)	17 (4.9)	**0.004**
Mini International neuropsychiatric interview				
Substance Dependence, No. (%)	322 (53.7)	170 (48.5)	152 (44.0)	0.23
Alcohol Dependence, No. (%)	274 (39.3)	152 (43.5)	122 (35.1)	**0.02**

*HF* Housing First, *TAU* Treatment-as-usual, *SD* standard deviation, *IQR* interquartile range, *CGI* Clinical Global Impression scale, *MCAS* Multnomah Community Ability Scale, *SF-36* Medical Outcomes Study 36-iIem Short-Form Health Survey, *PCS* physical composite score, *MCS* mental composite score; *MOUD* Medications for Opioid Use Disorder, *MINI* Mini International neuropsychiatric interview

### Mortality rate

4.8% (*n* = 34) of the study participants died over the study period. As shown in Table 2, the mortality rate was 0.065 in HF group, with 23 deaths occurring within 2 years and 0.034 in the TAU group with 11 patients who died (*p* = 0.058)**.** People with “uncertain vital status” were 33 (9.5%) in the TAU group and 2 (0.6%) in the HF group.

**Table 2:** Vital status of Participants within two years of follow up (*N =* 703)

	Total population	Deceased cases	Cases with Uncertain vital status	Mortality Rate at 24 months Estimate (Std error)	p
HF Group	353	23	2	0.065 (0.013)	0.058
TAU Group	350	11	33	0.034 (0.010)	

*HF* Housing First, *TAU* Treatment-as-usual, *Std. Error* standard error

The mean age of deceased individuals was 40.9 (**±** 11.4) years, 85.3% were men and 65% were diagnosed with schizophrenia.

### Survival analysis

**Fig. 1:**
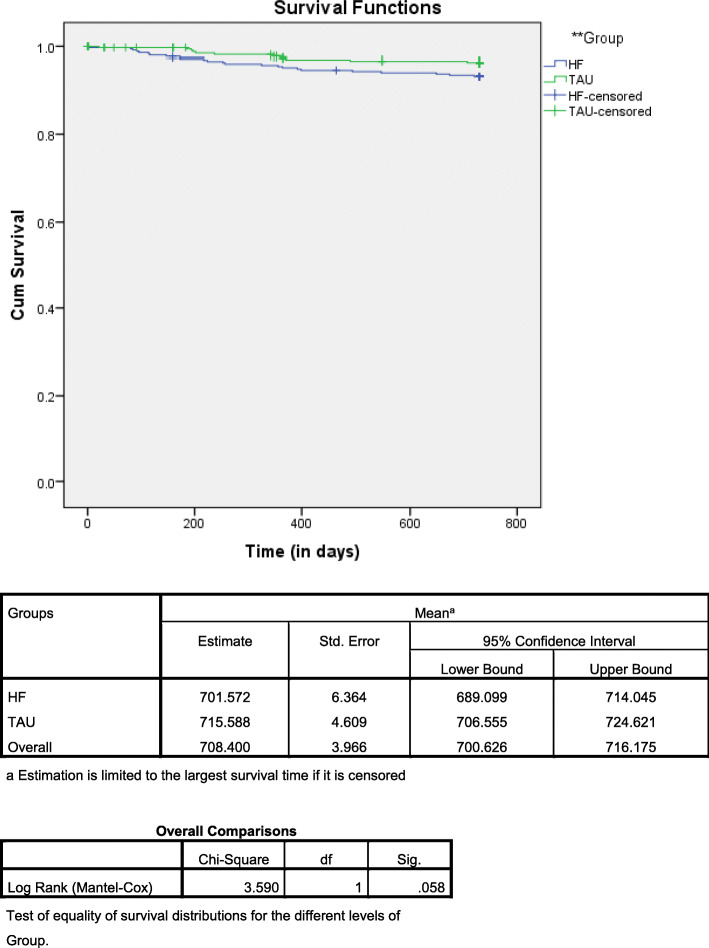
Kaplan Meier survival analysis

Mean survival time was 1.92 years (95%CI: 1.89–1.96) in the HF group and 1.96 years (95%CI: 1.93–1.98) in the TAU group (*P* = 0.058) (Fig. 1). In the HF group, 39% of deaths occurred within the first 6 months following entry into housing, compared to 18% in the TAU group.

In the Cox regression model, only one factor turned out to be significant predictors: taking MOUD (HR 2.37, 95%CI 1.11–5.05 (Table 3). (Table 3). The variable “group” was just barely below the level of significance (*P* = 0.054) with those in the HF group having almost a three-fold increase (HR: 0.49, 95%CI 0.24–1.01) in the risk of death compared to the TAU group.

**Table 3:** Death differences in mental and physical health comorbidities and self-reported health status and quality of life between the 34 deceased homeless and the 669 alive homeless ^a^ population with schizophrenia (SZ) or bipolar disorders (BD)

Variables	***Univariate Cox analysis*** ***HR*** ***(95%CI)***	***p***	***Multivariate Cox analysis*** ***HR*** ***(95%CI)***	***p***
Group (HF vs. TAU)	0.58 (0.28–1.19)	0.141	0.49 (0.24–1.01)	0.054^b^
Gender (Men vs. Women)	0.89 (0.35–2.31)	0.816		
Age	1.02 (0.99–1.05)	0.250		
Education, (Bac vs. <bac)	0.99 (0.95–1.05)	0.976		
Housing status, (Abs. vs. Prec.)	1.09 (0.53–2.23)	0.821		
Lifetime duration of homelessness	0.85 (0.41–1.78)	0.662		
Mental diagnosis (SZ vs. BD)	1.26 (0.62–2.54)	0.521		
CGI Score	1.03 (0.78–1.35)	0.854		
MCAS score	0.97 (0.92–1.01)	0.164		
PCS SF-36 score	0.97 (0.95–1.01)	0.084		
MCS SF-36 score	0.99 (0.95–1.01)	0.922		
Violent victimization	1.13 (0.55–2.20)	0.739		
Nonviolent victimization	2.02 (0.96–4.24)	0.062		
Alcohol dependence	1.50 (0.61–3.70)	0.376		
Substance dependence	1.02 (0.40–2.59)	0.965		
Hospitalization	1.28 (0.95–1.73)	0.097		
Taking medicines	1.33 (0.58–3.08)	0.499		
Medications for Opioid Use Disorder	2.31 (1.08–4.91)	**0.030**	2.37 (1.11–5.05)	**0.025**
Psychotropic drugs	0.98 (0.47–2.05)	0.972		
Other treatments	0.98 (0.47–2.05)	0.972		
−2 Log Likelihood		Block 0	441.742	
		Block 1	434.195	0.018

^a^Alive at the date of the last follow-up visit or the date of the last news for those who were initially lost to follow-up

^b^After controlling for all interactions between variable “group” and covariates, none interaction were kept in the final model

Values in bold indicate a statistically significant difference between groups

*HF* Housing First, *TAU* Treatment-as-usual, *Abs. vs; Pres.* Absence or Presence, *CGI* Clinical Global Impression scale, *MCAS* Multnomah Community Ability Scale, *SZ* schizophrenia, *BD* bipolar disorder, *SF-36* Medical Outcomes Study 36-iIem Short-Form Health Survey, *PCS* physical composite score, *MCS* mental composite score, *MOUD* Medications for Opioid Use Disorder

### Causes of death

Among those who died in the whole study, 14 of the 34 patients (41.2%) developed a cancer, an infection, died from a cardiovascular or a liver disease, or other natural cause (Table 4), while violent deaths by suicides or accidents (including overdoses) were reported in 14 patients (41.2%).

**Table 4:** Comparison of causes of death observed among HF and TAU participants

	Deaths in HF group ***N = 23***	Deaths in TAU group ***N = 11***	Total deaths ***N = 34***
**Causes of death**	*N (%)*	*N (%)*	*N (%)*
**Violent deaths / external**	**12 (52.2%)**	**2 (18.2%)**	**14 (41.2%)**
Suicides	3 (13.1%)	1 (9.1%)	4 (11.7%)
Accidents other than overdoses	1 (4.3%)	1 (9.1%)	2 (5.9%)
Overdoses of drugs	8 (34.8%)	0	8 (22.8%)
**Natural deaths / internal**	**9 (39.1%)**	**5 (45.4%)**	**14 (41.2%)**
Cardiovascular diseases	2 (8.7%)	2 (18.2%)	4 (11.7%)
Cancers	3 (13.1%)	1 (9.1%)	4 (11.7%)
Infections	2 (8.7%)	1 (9.1%)	3 (8.8%)
Liver diseases (cirrhosis including)	1 (4.3%)	0	1 (2.9%)
Other natural death	1 (4.3%)	1 (9.1%)	2 (5.9%)
**Undetermined causes**	**2 (8.7%)**	**4 (36.4%)**	**6 (17.6%)**

Undetermined causes of deaths represented 17.6% of all deaths, with 8.7% in HF group versus 36.4% in TAU group (p (Fisher) = 0.07).

Among known causes of death, external causes (e.g. suicides and accidents including overdoses) represented 52.2% of the deaths in the HF group and 18.2% in the TAU group (*P* Fisher = 0.08).

An overdose of drugs was the main causes of death for 8 patients and was exclusive in the HF group when compared to TAU group (34.8% vs. 0%, *P* = 0.02). No significant differences were found according to the other causes of death between the 2 groups.

## Discussion

This is the first randomized controlled trial on Housing First that examined and compared mortality rate and its causes. Our results can be summarized as follows: i) in only 2 years, almost 5% of deaths were observed for this rather young cohort (38.8 years at inclusion) of homeless persons with schizophrenia or bipolar disorders, with an average age at death of 40.9 years, ii) although the difference did not reach significance, there was an excess mortality in the HF group, mainly during the first 6 months of observation, iii) drug overdose was the leading cause of death in the HF group, accounted for one-third of deaths versus none in the TAU group and iv) in multivariate analysis mortality was associated with the use of MOUD.

### Long-term effects of homelessness and risk persistence

Our results support the findings of several authors that homelessness is not only an independent risk factor of mortality, but also a persistent one over time. Stenius-Ayoade followed a large cohort of homeless people after shelter use in Helsinki (mean age: 49.4 years), and showed that 10 years after 52% were deceased, versus 14.6% of the age-matched control group, even if “only” 5% were still homeless [30]. For survivors, Oppenheimer showed that a history of homelessness (i.e. have been homeless in the past) remains significantly associated with negative outcomes as economic precariousness, engagement in high-risk health behaviours, and worse mental health and physical health, even after controlling for multiple risk factors and social assets [31]. Henwood et al. which conducted the most similar study of the current one (focused on homeless people with severe mental disorders rehoused in Housing First program but without control group) showed that the risk of death was still higher in this population than the general population, with RR 4.4 for male HF participants [32]. Interestingly, Henwood find the period of the first 6 months in housing to be the period most at risk of death, which we also find in our study results. HF participants cumulate risk factors of premature mortality - schizophrenia or bipolar disorders, addiction, high-needs of service - and this should encourage HF teams to be particularly attentive during this 6-months period.

### Non-significant excess mortality in HF group

Our results suggesting excess mortality in the HF group versus the TAU group - without the difference reaching the threshold of significance - must be tempered from the outset with important limitations. Firstly, 2 people (8.7%) in HF group died before HF intervention, but their deaths were analyzed in intention-to-treat according to the data analysis plan because they occurred after randomization. Then the current comparison was not between newly housed and un-housed people who were homeless with psychotic disorders (per protocol) but between randomization groups, regardless of allocation. Secondly, no proof of life or death in any of the data sources has been found for only 0.6% in the HF group (*n* = 2) but for 9.5% people in the TAU group (*n* = 33). These missing persons - who gave no sign of life to their family and friends, did not generate any trace in social and medical information systems and administrative databases - could be people changing countries but also unidentified deceased persons. If so, the number of deaths in the TAU group could be underestimated. In France, the number of unidentified decedents is around 1000–3000 annually, including many homeless people [33]. This number of unidentified deaths is much higher than in other European countries [34] due to legal provisions as well as the absence of a dedicated database [33]. Homeless individuals are a group at higher risk of dying unidentified [35], with what authors called “social death” [36] following exclusion in life. Although NGOs such as ‘Morts de la Rue’ (Dead on the Streets) lead advocacy campains to raise awareness of the population and public authorities to the ethical issues at stake [37], their action remain mainly symbolic and too localized to have a significant impact on our data.

Finally, the third study limitation is the small number of events (deaths), which reduce the statistical power of the analysis. To take this low event scenario into account, we used *p* value < 0.05 to select variables for the multivariable model and we forced the group variable in the multivariable cox regression, with only one covariate being eligible at this significance level.

### Overdoses in HF group

Over the past decade, addictive behaviours in the HF programs has been evaluated and yielded mixed results [38]: i) no significant differences in alcohol and drug use has been shown between HF and TAU [39]; ii) traditional treatment was more effective in drug cessation [40]; or iii) HF participants had lower substance use [19, 41]. One of the results of the French HF trial was the absence of a difference in the addictive behaviors in the HF group in comparison with the TAU group, with a large decrease in both groups over the 2 years of follow up [14]. HF is a harm reduction approach, aiming to minimize risks even if the person remains addicted [42, 43]. As a consequence, this approach tolerates a certain level of risk behaviors [44]. In France, HF and TAU approach to addiction are very different, with strict prohibition of substance use in most places where TAU group spends nights (vast majority of shelters, medical or penal institutions), when HF does not coerce drug behavior, relying on a non-judgmental and recovery oriented approach. The population of the study accumulates mental illness, addiction and homelessness as well-known risk factors for overdoses, and the overdose mortality rate in our study is four times higher than in the general homeless population in France [6]. The finding that all identified overdoses are found in HF group is puzzling but may be not generalizable due to local poor clinical practices: for example, French HF teams of that time did not promote the use of naloxone emergency kits. This calls for a better training of HF teams focusing on harm reduction with targeted education on addiction issues and the risks of overdose [43, 45]. This result must be balanced by the large difference in the rates of undetermined causes in the two groups: 8.7% in HF versus 36.4% in TAU. The search for a cause and the identification of an overdose by the doctor in charge of the death certificate is influenced by the presence of an entourage, which may explain why the causes of death of homeless people are more often undetermined than those of the general population [12]. A home intensive support team like HF will provide more useful information to the coroner about the daily life of the deceased person, his or her relationship to medication and substance use. For people in TAU group, who do not benefit from such a support, the diagnosis of overdose may be underestimated.

### Medications for opioid use disorder (MOUD)

Across both groups of the present study, the use of MOUD (methadone or buprenorphine) was a predictor for lower survival rates. Yet, pharmaceutical interventions have proven to be effective in reducing mortality, morbidity and substance use in opioid use disorders [46–48], including among homeless population, but with a lower level of evidence [49]. France has the highest coverage of medications for opioid use disorder in Europe, with 80% of high-risk users receiving medications [50]. The rate of deaths directly related to drugs in France is one of the lowest in the European Union (4 to 6 per million inhabitants). Although partly due to an underestimation, this low rate of deaths also reflects the effect of the French policy of opioid addiction management [51], which presents some striking features. This policy relies on a primary care system where buprenorphine is prescribed by general practitioners and dispensed by retail pharmacies [52] in addition to a specialist services for the management of addiction with facilities called CSAPA and CAARUD, many of which have a methadone clinic activity [53]. To note, opiate overdose deaths have declined by approximately 80% in the 20 years following the introduction of buprenorphine delivery modalities in 1995 [54].

With regard to the accessibility for homeless population, France has a highly developed network of low-threshold centers (CAARUDs) which welcome more than 50% of people living in precarious conditions [55]. These low-threshold centers follow 75,000 people annually. The interest of buprenorphine’s accessibility in France has been particularly highlighted for marginalized populations and for complex situations, as lowering the threshold to access care in this population [56].

By contrast with these positive elements, naloxone was not easily accessible for users in the community and there were no supervised consumption use facilities in France during the period of the study. This kind of facility has been shown to reduce overdose [49] and our results suggest that this solution should be considered urgently to complete the provision of support for this vulnerable population, as well as the expansion of the access to naloxone.

In the current study, 14 among 34 deceased people had medications for opioid use disorder. All MOUD have a toxicity and can potentially cause death [57] but continued heroin use is associated with higher risks: mortality risks among opioid users during MOUD is less than a third of that expected in the absence of medications [48].

Buprenorphine, which is a partial opioid agonist, is considered a safer MOUD than methadone, which is a full agonist [57], but in our study, 7 of deceased people took buprenorphine (5 in HF group – 2 in TAU group), and 7 took methadone (4 in HF group - 3 in TAU group).

We lack precise data on the use of other medications, drugs, or recent history with MOUD (such as cessation and resumption) just prior to death but the time immediately after leaving medication with both drugs (methadone and buprenorphine) is a period of particularly increased mortality risk [48]. We hypothesize an association between use of psychotropic drugs, which account for the overwhelming majority of medical treatment used by the participants of the study, and overdoses. Indeed, an American study of 38,329 overdose death certificates showed the importance of frequently prescribed psychiatric drugs in overdoses: benzodiazepines (30.1%), antidepressants (13.4%), antiepileptic or antiparkinsonian drugs (6.8%), and antipsychotics and neuroleptics (4.7%) [58]. In addition, heavy therapeutic use and nonmedical use of prescription drugs have been described among homeless people, especially among the youngest [59], alongside misuse of prescribed substances, including voluntary drug poisoning [45, 60–62].

We recommend implementing a careful analysis of the mortality data in future HF studies, with special attention to use of other medications, drugs, cessation and resumptions in the period prior to death.

### Suicide

Deaths by suicide in this study were significantly higher than in the general French homeless population – 11.4% compared to 5% [12]. This is likely related to the diagnosis inclusion criteria for this RCT, given the noted high presence of psychiatric disorders in suicide cases [63, 64] which is particularly acute for homeless populations [10, 65]. Again, the inclusion criteria for HF program targeted some of the most vulnerable within the homeless population, often those with dual diagnosis of schizophrenia or bipolar disorders alongside homelessness and frequent substance addiction. To note, the identification of suicide among causes of deaths is influenced by the entourage’s knowledge of suicidal ideation and, as with overdoses, the daily presence of a team in the HF group probably helped the identification of causes better than in the TAU group. To differentiate between death by overdose with suicidal intention and unintentional death by overdose is particularly difficult.

### Internal causes

Our data showed that more than one-third of deaths were due to diseases in the *Un Chez Soi d’Abord* trial, with 11.7% from cancer. In France, the Epidemiology Centre on medical Causes of Death (CepiDC-Inserm) reported for the year 2016 34% deaths related to cancer [66] The higher rate of cancer-related deaths in the HF group is likely to result from higher levels of diagnosis and treatment in this group than the TAU group.

To conclude, our results confirm that the vulnerability of long-term homeless people with SMI continues after access to independent housing and suggest that better preventive approaches should be considered.

- From a medical perspective since HF participants are at high risk of opioid overdose, pros and cons of psychotropic drugs prescription must be carefully weighed up, with adequate education of service users about the risks, and increased availability of life-saving opioid antagonist like naloxone, which should be co-prescribed as often as necessary.
- At an institutional level harm reduction approaches and active specific support for addictions appears highly necessary for HF participants. In France, supervised drug consumption facilities should move from the experimental to the operational stage.
- At a policy level since the damage caused by homelessness is not easily reversible, public policies should more focus on primary prevention of homelessness, with earlier intervention in the path of homelessness.

## Data Availability

The datasets generated and analysed during the current study are not publicly available due to special authorization to transfer databases given by the CNIL. Upon prior authorization by the CNIL, the dataset would be available from corresponding author on reasonable request.

## Abbreviations

BD: Bipolar disorder
DSM-IV-TR: Diagnostic and Statistical Manual of Mental Disorders, fourth edition
HF: Housing First
MCAS: Multnomah Community Ability Scale
MCS: Mental composite score
MINI: Mini International Neuropsychiatric Interview
MOUD: Medications for Opioid Use Disorder
NGOs: Non-governmental organizations
PCS: Physical composite score
QoL: Quality of life
RCT: Randomized controlled trial
SCZ: Schizophrenia
SD: Standard deviation
SF-36: Medical Outcomes Study 36-item Short Form Health Survey
SMI: Severe mental illness

## References

[1] Ivers J-H, Zgaga L, O’Donoghue-Hynes B, Heary A, Gallwey B, Barry J (2019). Five-year standardised mortality ratios in a cohort of homeless people in Dublin. BMJ Open.

[2] Stenius-Ayoade A, Haaramo P, Kautiainen H, Gissler M, Wahlbeck K, Eriksson JG. Mortality and causes of death among homeless in Finland: a 10-year follow-up study. J Epidemiol Community Health. 2017:jech-2017-209166. 10.1136/jech-2017-209166. Epub ahead of print. 10.1136/jech-2017-209166 28739837

[3] Cheung AM, Hwang SW (2004). Risk of death among homeless women: a cohort study and review of the literature. CMAJ.

[4] Aldridge RW, Menezes D, Lewer D, Cornes M, Evans H, Blackburn RM, et al. Causes of death among homeless people: a population-based cross-sectional study of linked hospitalisation and mortality data in England. Wellcome Open Res [Internet]. 2019;4 [cité 28 févr 2020]. Disponible sur: https://www.ncbi.nlm.nih.gov/pmc/articles/PMC6449792/. 10.12688/wellcomeopenres.15151.1 PMC6449792 30984881

[5] Nusselder WJ, Slockers MT, Krol L, Slockers CT, Looman CWN, Beeck EF v (2013). Mortality and Life Expectancy in Homeless Men and Women in Rotterdam: 2001–2010. PLoS One.

[6] Montgomery AE, Szymkowiak D, Culhane D (2017). Gender differences in factors associated with unsheltered status and increased risk of premature mortality among individuals experiencing homelessness. Womens Health Issues.

[7] Hassanally K, Asaria M (2018). Homeless mortality data from East London. London J Prim Care.

[8] Slockers MT, Nusselder WJ, Rietjens J, van Beeck EF (2018). Homeless adults’ most frequent cause of death is suicide or murder. Ned Tijdschr Geneeskd.

[9] Beijer U, Andreasson S, Agren G, Fugelstad A (2011). Mortality and causes of death among homeless women and men in Stockholm. Scand J Public Health.

[10] Nielsen SF, Hjorthøj CR, Erlangsen A, Nordentoft M (2011). Psychiatric disorders and mortality among people in homeless shelters in Denmark: a nationwide register-based cohort study. Lancet.

[11] Feodor Nilsson S, Hjorthøj CR, Erlangsen A, Nordentoft M (2014). Suicide and unintentional injury mortality among homeless people: a Danish nationwide register-based cohort study. Eur J Pub Health.

[12] Vuillermoz C, Aouba A, Grout L, Vandentorren S, Tassin F, Vazifeh L (2014). Estimating the number of homeless deaths in France, 2008–2010. BMC Public Health.

[13] Aldridge RW, Story A, Hwang SW, Nordentoft M, Luchenski SA, Hartwell G (2018). Morbidity and mortality in homeless individuals, prisoners, sex workers, and individuals with substance use disorders in high-income countries: a systematic review and meta-analysis. Lancet.

[14] Tinland A, Loubière S, Boucekine M, Boyer L, Fond G, Girard V, et al. Effectiveness of a housing support team intervention with a recovery-oriented approach on hospital and emergency department use by homeless people with severe mental illness: a randomised controlled trial. Epidemiol Psychiatric Sci [Internet]. 2020;29 [cité 5 oct 2020]. Disponible sur: https://www.cambridge.org/core/journals/epidemiology-and-psychiatric-sciences/article/effectiveness-of-a-housing-support-team-intervention-with-a-recoveryoriented-approach-on-hospital-and-emergency-department-use-by-homeless-people-with-severe-mental-illness-a-randomised-controlled-trial/4EFD852DDA12E45E9516D9AC801D1682/core-reader. 10.1017/S2045796020000785 PMC7576524 32996442

[15] Aubry T, Goering P, Veldhuizen S, Adair CE, Bourque J, Distasio J (2015). A Multiple-City RCT of Housing First With Assertive Community Treatment for Homeless Canadians With Serious Mental Illness. PS.

[16] Stergiopoulos V, Hwang SW, Gozdzik A, Nisenbaum R, Latimer E, Rabouin D (2015). Effect of Scattered-Site Housing Using Rent Supplements and Intensive Case Management on Housing Stability Among Homeless Adults With Mental Illness: A Randomized Trial. JAMA.

[17] Sadowski LS, Kee RA, VanderWeele TJ, Buchanan D (2009). Effect of a housing and case management program on emergency department visits and hospitalizations among chronically ill homeless adults: a randomized trial. JAMA.

[18] Henwood BF, Dichter H, Tynan R, Simiriglia C, Boermer K, Fussaro A (2015). Service use before and after the provision of scatter-site housing first for chronically homeless individuals with severe alcohol use disorders. Int J Drug Policy.

[19] Padgett DK, Stanhope V, Henwood BF, Stefancic A (2011). Substance use outcomes among homeless clients with serious mental illness: comparing housing first with treatment first programs. Community Ment Health J avr.

[20] Palepu A, Patterson ML, Moniruzzaman A, Frankish CJ, Somers J (2013). Housing first improves residential stability in homeless adults with concurrent substance dependence and mental disorders. Am J Public Health.

[21] Tinland A, Fortanier C, Girard V, Laval C, Videau B, Rhenter P (2013). Evaluation of the Housing First program in patients with severe mental disorders in France: study protocol for a randomized controlled trial. Trials.

[22] American Psychiatric Association (2000). Diagnostic and Statistical Manual of Mental Disorders, 4th Edition text revised. (DSM-IV-TR).

[23] Barker S, Barron N, McFarland BH, Bigelow DA. A community ability scale for chronically mentally ill consumers: part I. Reliability and validity Community Ment Health J août 1994;30(4):363–383, DOI: 10.1007/BF02207489. 10.1007/BF02207489 7956112

[24] Ware JE, Sherbourne CD (1992). The MOS 36-item short-form health survey (SF-36). I Conceptual framework and item selection. Med Care.

[25] Leplège A, Ecosse E, Verdier A, Perneger TV (1998). The French SF-36 Health Survey: Translation, Cultural Adaptation and Preliminary Psychometric Evaluation. J Clin Epidemiol.

[26] Sheehan DV, Lecrubier Y, Sheehan KH, Amorim P, Janavs J, Weiller E (1998). The Mini-international neuropsychiatric interview (M.I.N.I): the development and validation of a structured diagnostic psychiatric interview for DSM-IV and ICD-10. J Clin Psychiatry.

[27] Amore K, Baker M, Howden-Chapman P (2011). The ETHOS Definition and Classification of Homelessness: An Analysis.

[28] van Buuren S (2007). Multiple imputation of discrete and continuous data by fully conditional specification. Stat Methods Med Res.

[29] Karim ME, Gustafson P, Petkau J, Tremlett H, Long-Term Benefits and Adverse Effects of Beta-Interferon for Multiple Sclerosis (BeAMS) Study Group (2016). Comparison of Statistical Approaches for Dealing With Immortal Time Bias in Drug Effectiveness Studies. Am J Epidemiol.

[30] Stenius-Ayoade A, Haaramo P, Kautiainen H, Sunikka S, Gissler M, Wahlbeck K (2018). Morbidity and housing status 10 years after shelter use-follow-up of homeless men in Helsinki, Finland. Eur J Pub Health.

[31] Oppenheimer SC, Nurius PS, Green S (2016). Homelessness history impacts on health outcomes and economic and risk behavior intermediaries: new insights from population data. Fam Soc.

[32] Henwood BF, Byrne T, Scriber B (2015). Examining mortality among formerly homeless adults enrolled in Housing First: An observational study. BMC Public Health.

[33] Malfroy Camine L, Schuliar Y, De Trane C, Kaempf C, Hutt J-M (2015). Personnes recherchées et « Enterrés sous X » : projet d’harmonisation des fichiers d’identification. La Revue de Médecine Légale.

[34] Cattaneo C, Ritz-Timme S, Schutz HW, Collins M, Waite E, Boormann H, Grandi M, Kaatsch HJ (2000). Unidentified cadavers and human remains in the EU: an unknown issue. Int J Legal Med.

[35] Paulozzi LJ, Cox CS, Williams DD, Nolte KB (2008). John and Jane doe: the epidemiology of unidentified decedents. J Forensic Sci.

[36] Parra RC, Anstett É, Perich P, Buikstra JE. Unidentified deceased persons. In: forensic science and humanitarian action [internet]: John Wiley & Sons, ltd; 2020. p. 79–99. [cité 1 déc 2020]. Disponible sur: https://onlinelibrary.wiley.com/doi/abs/10.1002/9781119482062.ch6

[37] Terrolle D (2002). La mort des SDF à Paris : un révélateur social implacable. Etudes sur la mort.

[38] Woodhall-Melnik JR, Dunn JR. A systematic review of outcomes associated with participation in Housing First programs. Hous Stud. 2016;31(3):287–304.

[39] Padgett DK, Gulcur L, Tsemberis S. Housing First Services for People Who Are Homeless With Co-Occurring Serious Mental Illness and Substance Abuse. Res Social Work Pract [Internet]. 2016; [cité 30 oct 2020]; Disponible sur: https://journals.sagepub.com/doi/10.1177/1049731505282593.

[40] Westermeyer J, Lee K (2013). Residential Placement for Veterans With Addiction: American Society of Addiction Medicine Criteria vs. a Veterans Homeless Program. J Nerv Ment Dis.

[41] Tsemberis S, Kent D, Respress C (2012). Housing stability and recovery among chronically homeless persons with co-Occuring disorders in Washington. DC Am J Public Health.

[42] Tsemberis S, Gulcur L, Nakae M (2004). Housing first, consumer choice, and harm reduction for homeless individuals with a dual diagnosis. Am J Public Health.

[43] Watson DP, Shuman V, Kowalsky J, Golembiewski E, Brown M. Housing First and harm reduction: a rapid review and document analysis of the US and Canadian open-access literature. Harm Reduct J [Internet]. 2017;14 [cité 22 nov 2019]. Disponible sur: https://www.ncbi.nlm.nih.gov/pmc/articles/PMC5442650/. 10.1186/s12954-017-0158-x PMC5442650 28535804

[44] Wenzel SL, Rhoades H, La Motte-Kerr W, Duan L, Harris T, Rice E (2019). Do HIV risk and prevention behaviors change over time among adults in permanent supportive housing?. AIDS Care.

[45] Bauer LK, Brody JK, León C, Baggett TP (2016). Characteristics of homeless adults who died of drug overdose: a retrospective record review. J Health Care Poor Underserved.

[46] Mattick RP, Kimber J, Breen C, Davoli M (2008). Buprenorphine maintenance versus placebo or methadone maintenance for opioid dependence. Cochrane Database Syst Rev.

[47] Mattick RP, Breen C, Kimber J, Davoli M (2009). Methadone maintenance therapy versus no opioid replacement therapy for opioid dependence. Cochrane Database Syst Rev.

[48] Sordo L, Barrio G, Bravo MJ, Indave BI, Degenhardt L, Wiessing L, et al. Mortality risk during and after opioid substitution treatment: systematic review and meta-analysis of cohort studies. BMJ [Internet]. 2017:357 [cité 25 mai 2021]. Disponible sur: https://www.ncbi.nlm.nih.gov/pmc/articles/PMC5421454/. 10.1136/bmj.j1550 PMC5421454 28446428

[49] Magwood O, Salvalaggio G, Beder M, Kendall C, Kpade V, Daghmach W (2020). The effectiveness of substance use interventions for homeless and vulnerably housed persons: A systematic review of systematic reviews on supervised consumption facilities, managed alcohol programs, and pharmacological agents for opioid use disorder. PLoS One.

[50] European Monitoring Centre for Drugs and Drug Addiction (2017). Country drug report 2017: France. [Internet].

[51] Brisacier A-C, Palle C, Mallaret M (2019). Décès directement liés aux drogues - Evaluation de leur nombre en France et évolution récentes. Tendances.

[52] Poloméni P, Schwan R (2014). Management of opioid addiction with buprenorphine: French history and current management. Int J Gen Med.

[53] Guillou-Landreat M, Dany A, Challet-Bouju G, Laforgue E, Leboucher J, Benoit Hardouin J (2021). What Differs between Patients under Methadone and under Buprenorphine for Opioid Use Disorder (OUD) in Daily Clinical Practice in France? A Short Report. Int J Environ Res Public Health.

[54] Auriacombe M, Fatséas M, Dubernet J, Daulouède J-P, Tignol J (2004). French field experience with buprenorphine. Am J Addict.

[55] Toufik A, Cadet-Taïrou A, Janssen E, Gandilhon M (2018). Profils, pratiques des usagers de drogues - ENa-CAARUD. Résultats de l’enquête nationale 2006 réalisée auprès des « usagers » des Centres d’accueil et d’accompagnement à la réduction des risques.

[56] Stancliff S, Joseph H, Furst T, Fong C, Comer SD, Roux P (2012). Opioid maintenance treatment as a harm reduction tool for opioid-dependent individuals in NYC: the need to expand access to buprenorphine in marginalized populations. J Addict Dis.

[57] Toce MS, Chai PR, Burns MM, Boyer EW (2018). Pharmacologic treatment of opioid use disorder: a review of pharmacotherapy, adjuncts, and toxicity. J Med Toxicol.

[58] Zivanovic R, Milloy M, Hayashi K, Dong H, Sutherland C, Kerr T (2015). Impact of unstable housing on all-cause mortality among persons who inject drugs. BMC Public Health.

[59] Barman-Adhikari A, Hsu H-T, Brydon D, Petering R, Santa Maria D, Narendorf S (2019). Prevalence and correlates of nonmedical use of prescription drugs (NMUPD) among Young adults experiencing homelessness in seven cities across the United States. Drug Alcohol Depend.

[60] Baggett TP, Chang Y, Singer DE, Porneala BC, Gaeta JM, O’Connell JJ (2014). Tobacco-, Alcohol-, and Drug-Attributable Deaths and Their Contribution to Mortality Disparities in a Cohort of Homeless Adults in Boston. Am J Public Health.

[61] Fazel S, Geddes JR, Kushel M (2014). The health of homeless people in high-income countries: descriptive epidemiology, health consequences, and clinical and policy recommendations. Lancet.

[62] Rayburn RL, Pals H, Wright JD (2012). Death, drugs, and disaster: mortality among New Orleans’ homeless. Care Manag J.

[63] Arsenault-Lapierre G, Kim C, Turecki G (2004). Psychiatric diagnoses in 3275 suicides: a meta-analysis. BMC Psychiatry.

[64] Cavanagh JTO, Carson AJ, Sharpe M, Lawrie SM (2003). Psychological autopsy studies of suicide: a systematic review. Psychol Med.

[65] Hwang SW, Wilkins R, Tjepkema M, O’Campo PJ, Dunn JR. Mortality among residents of shelters, rooming houses, and hotels in Canada: 11 year follow-up study. BMJ [Internet]. 2009;339 [cité 11 mars 2020]. Disponible sur: https://www-bmj-com.lama.univ-amu.fr/content/339/bmj.b4036. 10.1136/bmj.b4036 PMC2767481 19858533

[66] Breton D, Barbieri M, Belliot N, d’Albis H, Mazuy M (2019). L’évolution démographique récente de la France : une singularité en Europe ?. Population..

